# Direct nanodroplet and microbubble comparison for high intensity focused ultrasound ablation enhancement and safety

**DOI:** 10.1186/2050-5736-3-S1-O67

**Published:** 2015-06-30

**Authors:** Linsey Moyer, Kelsie Timbie, Paul Sheeran, Richard Price, Wilson Miller, Paul Dayton

**Affiliations:** 1 University of North Carolina, Chapel Hill, North Carolina, United States; 2 University of Virginia, Charlottesville, Virginia, United States; 3 Sunnybrook Health Sciences Centre, Toronto, Canada

## Background/introduction

High intensity focused ultrasound (HIFU) surgery often requires hours of ablation in order to treat an entire tumor. Both perfluorocarbon gaseous microbubbles and vaporized liquid droplets are known enhancers of HIFU thermal ablation. Microbubbles, however, often lead to surface or skin lesions. Furthermore, they have a relatively short half-life *in vivo* (minutes) rendering them insufficiently stable for an entire HIFU surgery, which can last several hours. Many droplet formulations require very high pressures to activate. Our aim was to design an agent that could shorten ablation procedures without sacrificing safety. We designed and investigated a perfluorocarbon nanodroplet composed of a 1:1 ratio of dodecafluoropentane and decafluorobutane. These are tuned to change phase and activate at only 2 MPa peak negative pressure with common HIFU pulse lengths, enabling focused and targeted activation. Additionally, they are stable at body temperature.

## Methods

Two lipid-shelled agents, microbubbles (2.1 +/-0.5 micron diameter) and nanodroplets (240 +/-65 nm diameter), were manufactured in-house. Effective circulation time of the nanodroplets was investigated *in vivo*. HIFU-induced temperature rises were measured as a function of the time after the injection of the agent occurred and were compared to controls wherein no agent was injected. HIFU (1 MHz, 4.06 MPa, CW, 15 seconds) was applied *in vivo* to rat livers 5, 15 or 95 minutes after agent injection, and any thermal enhancement was detected simultaneously by MR thermometry.

## Results and conclusions

**Figure 1:**
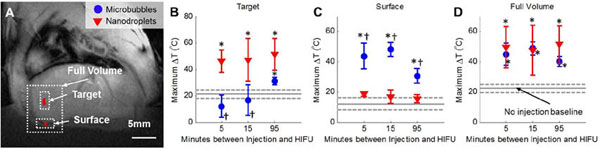
MR-guided thermometry of HIFU ablation (15 seconds at 15 Watts) of rat liver following the injection of nanodroplet or microbubble contrast agents. A) the hermometry map was divided into three regions for analysis: B) the target (focus) of the HIFU beam, C) the surface of the animal, and D) the full treated volume. The peak temperature change was calculated for each animal. The gray solid line indicated the mean temperature changed observed when HIFU was applied without an injection of contrast agents, and the dotted lines indicate one standard deviation, *indicates significance (p<0.05) compared to the baseline temperature rise (gray line), †indicates significance (p<0.05) between microbubbles and nanodroplets. (n=3 or 4, except for the 5 minute nanodroplet time point where n=2, data is displayed as the mean +/- S.D.)

HIFU applied to livers without any agent induced only a 22 +/-3ºC maximal temperature rise over body temperature. The maximum HIFU-induced temperature rise with microbubbles (55 +/-7 ºC) was observed 5 minutes after their injection, whereas nanodroplets consistently enhanced the HIFU thermal ablation at every time point, with a peak temperature change 95 minutes after their injection of 51 +/-12ºC (see Fig. 1). More importantly, the location where this heating occurred was vastly different between the two agents. Microbubbles primarily heated the surface of the animal and resulted in skin burns, whereas no skin burns were present in any of the control HIFU animals or those that received nanodroplet injections with HIFU. Nanodroplets resulted in HIFU heating at the target location with only minimal surface heating that was not significantly different from the baseline heating observed with HIFU alone (grey line in Fig. 1B, 1C, 1D). These results suggest that the nanodroplets are sufficiently stable to enhance HIFU ablation *in vivo* for at least 1.5 hours, avoid skin burns, and are a better option over microbubbles. These nanodroplets could potentially reduce focused ultrasound surgical procedure times by as much as 5 fold by more quickly ablating a larger region of tissue, without compromising safety.

